# Synthetic 3D Spinal Vertebrae Reconstruction from Biplanar X-rays Utilizing Generative Adversarial Networks

**DOI:** 10.3390/jpm13121642

**Published:** 2023-11-24

**Authors:** Babak Saravi, Hamza Eren Guzel, Alisia Zink, Sara Ülkümen, Sebastien Couillard-Despres, Jakob Wollborn, Gernot Lang, Frank Hassel

**Affiliations:** 1 Department of Orthopedics and Trauma Surgery, Medical Center—University of Freiburg, Faculty of Medicine, University of Freiburg, 79106 Freiburg, Germany; sara.uelkuemen@jupiter.uni-freiburg.de (S.Ü.); gernot.michael.lang@uniklinik-freiburg.de (G.L.); 2 Department of Spine Surgery, Loretto Hospital, 79100 Freiburg, Germany; alisia.zink@gmail.com (A.Z.); frank.hassel@rkk-klinikum.de (F.H.); 3 Institute of Experimental Neuroregeneration, Spinal Cord Injury and Tissue Regeneration Center Salzburg (SCI-TReCS), Paracelsus Medical University, 5020 Salzburg, Austria; s.couillard-despres@pmu.ac.at; 4 Department of Anesthesiology, Perioperative and Pain Medicine, Brigham and Women’s Hospital, Harvard Medical School, Boston, MA 02115, USA; jwollborn@bwh.harvard.edu; 5 Department of Radiology, University of Health Sciences, Izmir Bozyaka Training and Research Hospital, Izmir 35170, Türkiye; hamzaerenguzel@gmail.com; 6 Austrian Cluster for Tissue Regeneration, 1200 Vienna, Austria

**Keywords:** computed tomography, generative adversarial networks, deep learning, 3D reconstruction, spinal imaging, spinal diagnosis, spine surgery, quantitative measurement, clinical application

## Abstract

Computed tomography (CT) offers detailed insights into the internal anatomy of patients, particularly for spinal vertebrae examination. However, CT scans are associated with higher radiation exposure and cost compared to conventional X-ray imaging. In this study, we applied a Generative Adversarial Network (GAN) framework to reconstruct 3D spinal vertebrae structures from synthetic biplanar X-ray images, specifically focusing on anterior and lateral views. The synthetic X-ray images were generated using the DRRGenerator module in 3D Slicer by incorporating segmentations of spinal vertebrae in CT scans for the region of interest. This approach leverages a novel feature fusion technique based on X2CT-GAN to combine information from both views and employs a combination of mean squared error (MSE) loss and adversarial loss to train the generator, resulting in high-quality synthetic 3D spinal vertebrae CTs. A total of n = 440 CT data were processed. We evaluated the performance of our model using multiple metrics, including mean absolute error (MAE) (for each slice of the 3D volume (MAE0) and for the entire 3D volume (MAE)), cosine similarity, peak signal-to-noise ratio (PSNR), 3D peak signal-to-noise ratio (PSNR-3D), and structural similarity index (SSIM). The average PSNR was 28.394 dB, PSNR-3D was 27.432, SSIM was 0.468, cosine similarity was 0.484, MAE0 was 0.034, and MAE was 85.359. The results demonstrated the effectiveness of this approach in reconstructing 3D spinal vertebrae structures from biplanar X-rays, although some limitations in accurately capturing the fine bone structures and maintaining the precise morphology of the vertebrae were present. This technique has the potential to enhance the diagnostic capabilities of low-cost X-ray machines while reducing radiation exposure and cost associated with CT scans, paving the way for future applications in spinal imaging and diagnosis.

## 1. Introduction

Computed tomography (CT) scans are widely used in medical imaging due to their high-resolution and detailed insights into the internal anatomy of patients [1]. In the field of spinal imaging, CT scans play a critical role in the diagnosis and management of various spinal disorders, providing accurate information on bone structure, alignment, and pathological changes [2]. However, cumulative radiation exposure for patients, particularly in the context of repeated diagnostic procedures, is a concern. The approximate effective radiation dose for CT spine scans ranges from 2 to 11 mSv, with an average dose of 8.8 mSv [3,4,5]. Additionally, the cost of CT scans can be prohibitive, limiting their accessibility for healthcare providers and patients in resource-constrained settings [6].

Conventional X-ray imaging is associated with lower radiation exposure and cost compared to CT scans, making it a more accessible imaging modality. The approximate effective radiation dose for biplanar lumbar X-ray is between 0.8 to 1.8 mSv, with an average dose of 1.4 mSv [3,5]. However, X-ray images are inherently two-dimensional (2D) and may not provide the same level of anatomical detail as 3D CT scans, particularly when it comes to evaluating spinal vertebrae structures [3]. Thus, there is a growing interest in developing novel techniques that can leverage the advantages of X-ray imaging while providing 3D information akin to CT scans [1].

In recent years, multimodal data techniques and 3D reconstruction have gained significant attention in the field of medical imaging, particularly for their potential to improve diagnosis, treatment planning, and patient outcomes [7,8]. The ability to generate 3D representations of anatomical structures from 2D images offers several benefits, some of which are outlined below [9,10,11]:

(1) Enhanced visualization and interpretation: 3D reconstruction provides a more comprehensive view of complex anatomical structures compared to traditional 2D imaging. This enhanced visualization enables healthcare professionals to better understand the spatial relationships between different structures, leading to more accurate diagnoses and improved decision-making.
(2) Improved surgical planning and navigation: In the context of spinal surgery, 3D reconstructions can facilitate preoperative planning by allowing surgeons to assess the extent of spinal deformities or pathological conditions, as well as the ideal surgical approach. Additionally, 3D reconstructions can be used intraoperatively to guide surgical navigation, thereby increasing the precision and safety of the procedure.
(3) Patient-specific modeling and simulation: 3D reconstructions can be employed to create patient-specific models of spinal structures, which can be used for biomechanical analyses, finite element simulations, or personalized implant design. These patient-specific models may contribute to the development of more effective and personalized treatment strategies, ultimately improving patient outcomes.
(4) Enhanced patient communication and education: 3D reconstructions can facilitate communication between healthcare providers and patients by providing a more intuitive understanding of the patient’s condition, the proposed treatment plan, and the expected outcomes. This improved communication can lead to better patient engagement, satisfaction, and adherence to treatment recommendations.
(5) Reduced radiation exposure: By generating 3D reconstructions from a limited number of 2D X-ray images, the proposed technique has the potential to reduce the cumulative radiation dose associated with traditional CT scans. This reduction in radiation exposure is particularly important for patients who require repeated imaging over time, such as those with progressive spinal disorders or those undergoing long-term follow-up after surgical intervention.
(6) Cost-efficiency and availability: CT scanners frequently have a higher price tag compared to X-ray machines, which can make them less accessible in resource-limited settings or developing countries.

In summary, 3D reconstruction offers numerous benefits in the field of spinal imaging, with the potential to improve diagnostic accuracy, facilitate surgical planning, enable patient-specific modeling and simulation, enhance patient communication, reduce radiation exposure, and improve cost-effectiveness and availability in resource-limited settings.

**Figure 1:**
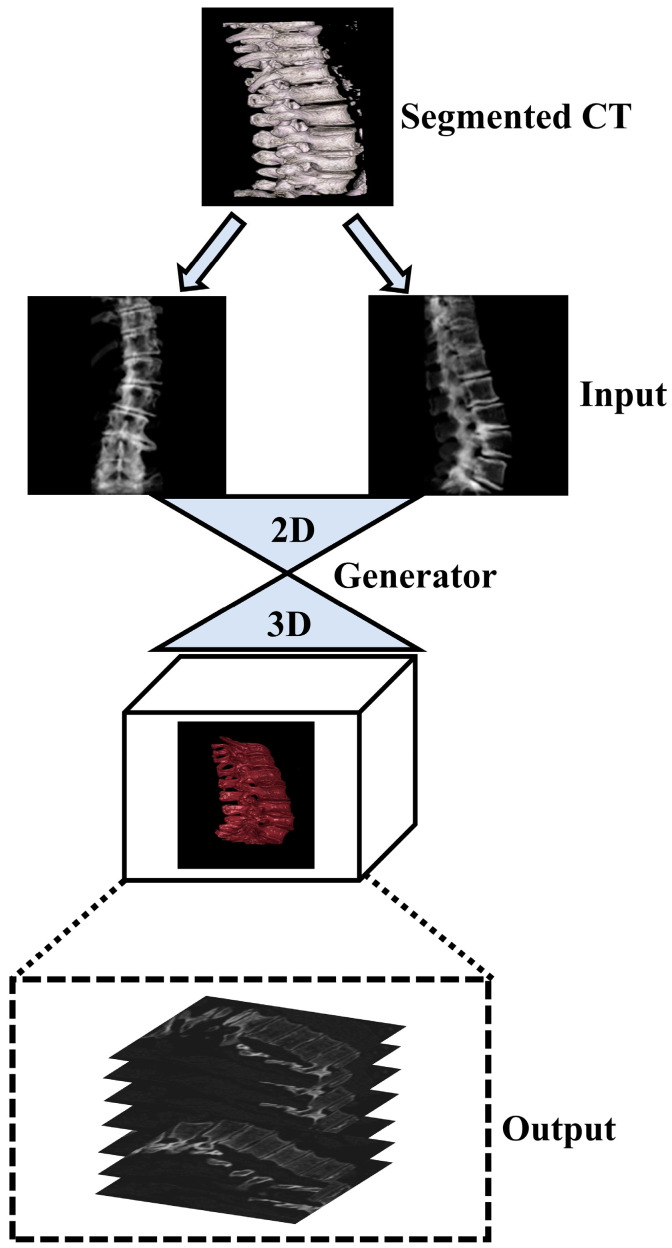
Illustration of the general concept for the synthetic 3D model generation of the spinal vertebrae based on biplanar X-ray input, where biplanar X-rays are focussed on the region of interest (spinal vertebrae) based on an initial segmentation step of the ground truth CT.

Generative adversarial networks (GANs) have emerged as powerful deep learning tools capable of synthesizing realistic images from different modalities. Recent studies have demonstrated the potential of GANs for generating 3D images from chest 2D X-ray projections, thus bridging the gap between conventional X-ray imaging and CT scans [11]. In this study, we applied the GAN-based framework for reconstructing 3D spinal vertebrae structures from synthetic biplanar X-ray images as presented by Ying et al. (X2CT-GAN), focusing specifically on anterior and lateral views of spinal vertebrae [11]. In contrast to previous work [11,12] which applied X2CT-GAN for 3D reconstruction in medical settings, we focussed on segmented vertebrae to specifically focus on the region of interest (spinal vertebrae) while reducing unnecessary information and thus computational cost. This approach leverages a novel feature fusion technique based on X2CT-GAN to combine information from both views and employs a combination of mean squared error (MSE) loss and adversarial loss to train the generator, resulting in high-quality synthetic 3D spinal vertebrae CTs. By incorporating a focused view of the spinal vertebrae through segmentation, this approach reduces unnecessary information which could affect the synthetic generation of CTs. The general concept is illustrated in Figure 1.

## 2. Materials and Methods

### 2.1. Dataset and Segmentation

In this study, we employed a COLONOG subset [13] of the CTSpine1K dataset [14], which is a large and comprehensive collection of spine CT data with segmentation masks and includes data of females and males > 50 years old. Given the focus of the original trial on colorectal screening, the CT scans were not explicitly aimed at spinal pathology diagnosis. Consequently, while the dataset does not contain explicit documentation of spinal pathologies, it can be inferred that the included patients were not primarily diagnosed with spinal conditions. The data can be obtained upon request from the authors of the CTSpine 1Kdataset (https://github.com/MIRACLE-Center/CTSpine1K; accessed on 15 August 2023). The prospective study to obtain the COLONOG subset was reviewed and approved by each participating institution’s institutional review board, and subjects gave their informed consent to participate and to have their private health information accessed for the purposes of the study. All data were anonymized when accessed through the database. A total of n = 440 CT data were processed. The data were acquired using multidetector-row CT scanners with a minimum of 16 rows while patients were in supine and prone positions. The CT images were obtained using the following specifications: collimation of 0.5 to 1.0 mm, pitch ranging from 0.98 to 1.5, a 512 by 512 matrix, and a field of view adjusted to fit the patient. The effective mAs was 50, and the peak voltage was 120 kV. A standard reconstruction algorithm was utilized, and images of patients in both prone and supine positions were reconstructed with slice thicknesses between 1.0 and 1.25 mm and a reconstruction interval of 0.8 mm [13].

As the benchmark, a fully supervised method was employed to train a deep network for spinal vertebrae segmentation using the nnUnet model [14]. The nnUnet model has outperformed other methods in various medical image segmentation tasks in recent years, making it the acknowledged baseline for medical image segmentation [15]. Essentially a U-Net, nnUnet features a specific network architecture and design parameters that self-adapt to the dataset’s characteristics, along with robust data augmentation [14,15]. The 3D full-resolution U-net architecture was used to accommodate the large volume of high-resolution 3D images in the dataset [14]. Further details about the nnUnet model can be found in the original publication [14].

To facilitate the annotation process, the segmentation network was trained using the public datasets from the VerSe’19 [16] and VerSe’20 Challenges [17], employing the nnUnet algorithm. As most of the samples from the VerSe’ Challenge were cropped, cases that had complete CT images and consistent image spacing between images and their corresponding ground truth were selected. Next, the trained segmentation model was assigned to junior annotators to predict segmentation masks and refine the labels based on the predictions. These refined labels were subsequently reviewed by two senior annotators for further improvement. In cases where the senior annotator encountered difficulties in determining the annotations, input was sought from experienced spine surgeons [14]. To ensure the final quality of the annotations, coordinators conducted a random double-check, and any incorrect cases were corrected by the annotators. The human-corrected annotations and corresponding images were added to the training data to develop a more powerful model. To expedite the annotation process, the database and retrained the deep learning model were updated after every 100 cases. This iterative process continued until the annotation task was completed. The entire annotation process was performed using ITK-SNAP software (version: 4.0.2), and segmentation masks were saved in NIfTI format [14].

### 2.2. Data Preprocessing and Synthetic X-ray Generation

In the initial stage of data preprocessing, we cropped the original CT data based on their respective segmentation masks (see Section 2.1). We implemented this procedure in Python by first defining a function that took two input arguments: the CT image and its corresponding segmentation mask. This function identified the non-zero elements in the segmentation mask and subsequently determined the minimum and maximum indices along the z, y, and x-axes. The function then returned the cropped CT data with dimensions ranging from the minimum to the maximum indices along each axis. We saved the cropped CT data as a new NIfTI file in the output directory with the same affine transformation as the original CT data.

Acquiring a sufficient number of original synchronized X-ray and CT images for machine learning purposes poses significant practical and ethical challenges. One major obstacle in training the X2CT-GAN model is the scarcity of paired X-ray and CT data from patients. Obtaining such paired data from patients is not only costly but also raises ethical concerns due to the additional radiation exposure involved. To address this issue, we have opted to train our network using synthesized X-rays generated from the ground truth CT dataset, as proposed before [11]. In this study, we employ a CT volume to simulate two X-rays corresponding to the posterior-anterior and lateral views. This process is achieved through the utilization of digitally reconstructed radiographs (DRR) technology [18]. By leveraging this approach, we can effectively generate the required paired X-ray and CT data for training the X2CT-GAN model without subjecting patients to unnecessary radiation risks. Consequently, the method strikes a balance between the need for accurate and diverse training data and the ethical concerns associated with obtaining such data from human subjects. To generate synthetic X-ray images from the cropped CT data, we used a custom-made Python script in the 3D Slicer software (version: 5.2.2.) (Python extension of 3D Slicer) [19]. For each subfolder containing the cropped CT data, the script modified the 3D viewer to display a black-white gradient and removed the bounding box and orientation axes labels. It then loaded the CT volume in 3D Slicer and switched to the one-up 3D view layout. The script defined a function which centered the 3D view and slice views to fill the background. This function was applied before setting up the volume rendering display. The volume rendering was set up by creating a default volume rendering display node and making it visible. The script then applied a “CT-X-ray” volume rendering preset from the DRRGenerator module [20] to the display node. To adjust the scalar opacity mapping, a six-point transfer function was defined and set as the scalar opacity for the volume property node. The function was called again to ensure that the view was centered. Next, the script rotated the 3D view and captured screenshots of the synthetic X-ray images in different orientations, such as the anterior and lateral views. The captured images were saved to disk in the respective subfolders as PNG files with appropriate filenames indicating the orientation. After processing each CT volume, the script cleared the 3D Slicer scene to prepare for the next iteration. By following this procedure, we generated synthetic X-ray images from the corresponding segmented CT data, which were then used for further analysis and experimentation.

**Figure 2:**
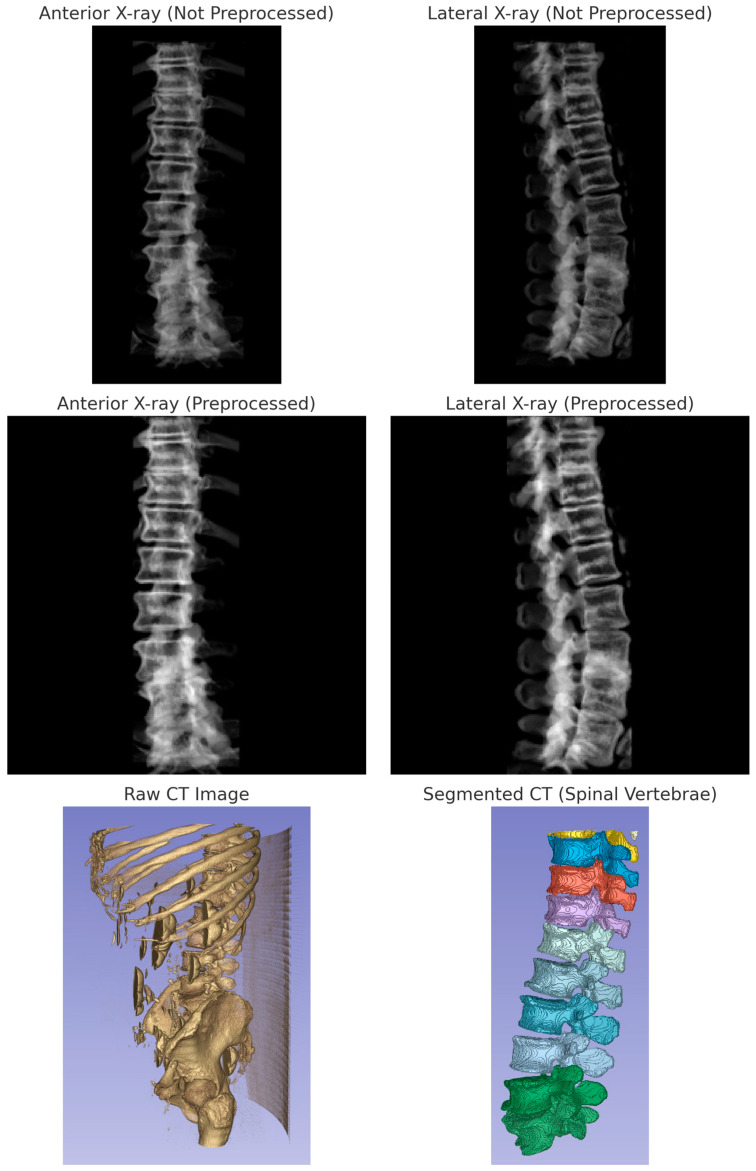
Multimodal imaging process and outputs. The top row displays the original biplanar X-ray images before preprocessing: anterior view and lateral view. The middle row shows the corresponding preprocessed X-ray images: anterior view and lateral view. The bottom row presents the raw CT image alongside the segmented CT reconstruction focusing on spinal vertebrae. These images represent the data pipeline from raw acquisition to segmentation and synthetic X-ray generation, underpinning the data preprocessing and analysis as outlined in Section 2.2 of the Materials and Methods.

The generated images were then preprocessed using the following method. A function was used to preprocess the synthetic X-ray images. This function takes an image as input and applies a series of image processing operations to it, including converting the image to grayscale, thresholding to keep only the brighter parts of the image, performing morphological operations to remove small noise and fill small gaps, finding the largest contour in the binary image, cropping the original image using the bounding box of the largest contour, normalizing the cropped image, padding the image to make it square, and resizing the padded image to the desired output size. The function was then applied to each synthetic X-ray image to produce a preprocessed image for the anterior and lateral view. Another function was implemented to load the CT images from NIfTI files. For each folder in the input folder, the code read in the preprocessed anterior and lateral X-ray images, as well as the CT image in NIfTI format. The preprocessed X-ray images and the CT image were then combined into a single HDF5 file for the current folder. The HDF5 file contained three datasets: “ct”, “xray1”, and “xray2”, corresponding to the CT image, synthetic anterior X-ray image, and synthetic lateral X-ray image, respectively. The data were then randomly split into a train set (80%) and a test set (20%) for further analyses. Figure 2 illustrates the image processing pipeline.

### 2.3. Model Training and Evaluation

**Figure 3:**
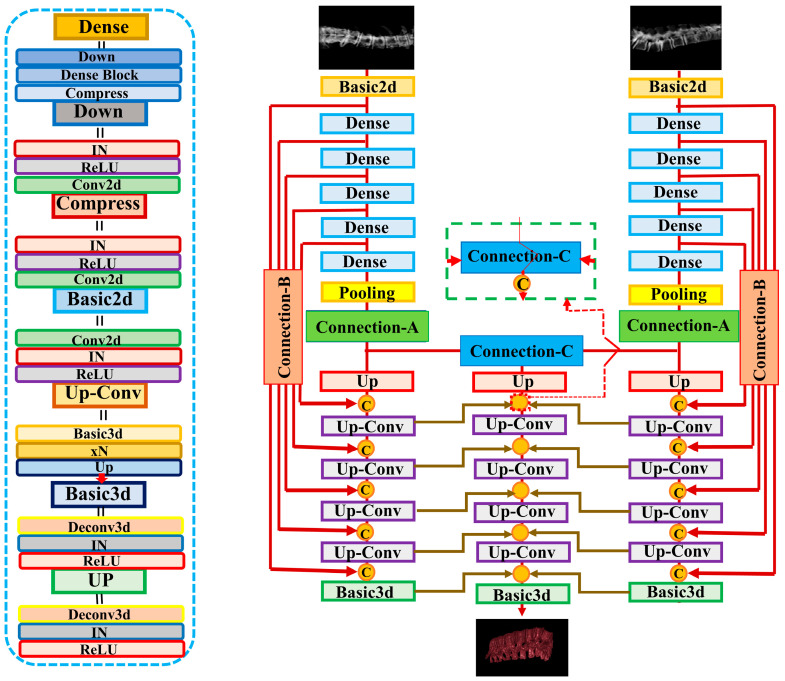
Illustration of the X2CT-GAN as proposed by Ying et al. [11]. In contrast to Ying et al. [11] who used unsegmented chest X-rays, we employed segmented biplanar X-ray inputs, resized to 128 × 128 pixels, and constructed 3D models with a focus specifically on the spinal vertebrae. The output dimensions of the reconstructed 3D CT models were 128 × 128 × 128 voxels with a voxel resolution of 1 mm × 1 mm × 1 mm. The generator network utilizes Basic2d convolution blocks to match channel dimensions and form pseudo-3D feature maps, which are then processed via Basic3d convolution blocks to encode the features into the 3D space of the decoder network.

In this study, we applied the X2CT-GAN model to fuse X-ray and CT images using a deep learning approach. The model was trained using a multiview network architecture consisting of a dense UNet fused with transposed convolutions as the generator and a basic 3D discriminator with instance normalization (Figure 3). The GAN loss was computed using least squares. The generator network employed ReLU activation functions and a conditional discriminator with no dropout layers. The model’s training parameters included a learning rate of 0.0002 (lr: 0.0002), Adam optimizer with beta1 set to 0.5, and beta2 set to 0.99. The training process employed a batch size of 1 due to limited computational resources (NVIDIA GTX 3090; 24 GB) and did not utilize weight decay. Data augmentation was applied to the images, with a fine size of 128 × 128, and images were resized to 150 × 150. The CT and X-ray images were normalized using the provided mean and standard deviation values. In the 3D reconstruction of spinal vertebrae from biplanar X-rays, the output dimensions of the reconstructed 3D CT models were 128 × 128 × 128 voxels with an output voxel size of 1 mm × 1 mm × 1 mm, ensuring a detailed representation of the anatomy with manageable data volumes for processing. Various loss functions and weighting parameters were used to optimize the model, such as identity loss, feature matching loss, map projection, and GAN. A detailed list of parameters, configurations, and functions used can be found in the code provided in the data availability section. During the training process, the discriminator and generator were optimized using the specified configuration parameters. The loss and metrics were logged, and the model was saved at specified intervals. The learning rate was updated at the end of each epoch.

For validation, the model was evaluated using a separate dataset, and the performance metrics were computed and logged. We used multiple metrics for evaluation to provide a sophisticated evaluation of the approach: (1) mean absolute error (MAE); (2) cosine similarity; (3) peak signal-to-noise ratio (PSNR); (4) structural similarity index measure (SSIM); (5) 3D peak signal-to-noise ratio (PSNR-3D).

First, the model and datasets were initialized, setting them to the evaluation mode in the X2CT-GAN model. We then iterated through the dataset, using the model to generate CT images. For each generated CT image, the metrics were calculated by comparing the generated CT image with the corresponding ground truth CT image. The metrics are as follows:

− Mean absolute error (MAE): MAE calculates the average absolute difference between the predicted and the ground truth CT images. It gives an idea of the magnitude of the errors without considering their direction. MAE was calculated for each slice (MAE0) and for the entire 3D volume (MAE).
− Cosine similarity: Cosine similarity measures the cosine of the angle between the predicted and the ground truth CT images, providing a similarity score between −1 and 1.
− Peak signal-to-noise ratio (PSNR): PSNR measures the ratio between the maximum possible power of a signal and the power of the noise.
− Structural similarity index measure (SSIM): SSIM measures the structural similarity between the predicted and the ground truth CT images.
− 3D peak signal-to-noise ratio (PSNR-3D): PSNR-3D is an extension of the PSNR metric for 3D images and measures the 3D image quality.

After calculating the metrics for each image, we computed the average value for each metric across the entire dataset. These average values represent the overall performance of the model using the specified evaluation metrics. Visualization was performed by importing the ground truth and synthetic CT data in 3D Slicer and using the CT-bone preset for visualization. Further, we implemented a comprehensive approach to evaluate the performance of the X2CT-GAN model under various imaging conditions. This included an analysis of the model’s sensitivity to changes in the angle between the anteroposterior and lateral biplanar X-ray images. Typically, biplanar X-rays are captured at a 90-degree orientation to each other, providing orthogonal views. To assess the robustness of the model against deviations from this standard setup, we conducted a series of analyses where the angle of the lateral X-ray image was adjusted relative to the anteroposterior image. Specifically, we analyzed the model’s performance with the lateral X-ray positioned at angles of 90 degrees (standard orthogonal), 85 degrees, 80 degrees, and 75 degrees in relation to the anteroposterior X-ray. We also incorporated a validation process to ascertain the fidelity of our synthetic X-ray generation and subsequent synthetic CT reconstruction. An original case with both biplanar X-ray and corresponding CT data was selected for this purpose. Synthetic X-rays were generated from the original CT data using 3D Slicer and our preprocessing pipeline, enabling a visual comparison with the actual biplanar X-rays to validate the synthetic image quality. Additionally, a synthetic CT was reconstructed from the original biplanar X-rays, employing the model trained on synthetic biplanar X-rays, to facilitate a comparison with the original CT data. This approach allowed us to critically evaluate the quality of both synthetic X-rays and CT reconstructions, providing insights into the training and performance limitations of the model. All analyses were performed using Python. The code for the custom Python scripts for 3D Slicer, the preprocessing scripts, and the configuration file is available from the data availability section. The original code of the X2CT-GAN is available from the repository provided by Ying et al. [11] and the modified code used for the present study is available from the data availability section.

## 3. Results

### 3.1. Quantitative Results

The evaluation of the X2CT-GAN model for generating synthetic CT scans based on biplanar X-ray inputs yielded promising results across various performance metrics. MAE and MSE were employed to assess the model’s accuracy. For the MAE per slice (MAE0), an average of 0.034 was recorded, while the MSE per slice (MSE0) obtained an average value of 0.005. The overall MAE and MSE were 85.359 and 31,439.027, respectively. Cosine similarity, which measures the angular distance between the generated synthetic CT scans and the ground truth, resulted in an average score of 0.484, indicating a reasonably good level of agreement between the two sets of data. PSNR is a metric that helps quantify the quality of the reconstructed image by comparing it to the original image. The average PSNR for the 3D reconstructed images (PSNR-3D) was 27.432. Furthermore, the PSNR values for individual channels, which can be interpreted as comparisons between the real and generated CT images in three different orientations or planes, were 28.817 (PSNR-1), 28.764 (PSNR-2), and 27.599 (PSNR-3), resulting in an average PSNR (PSNR-avg) of 28.394. Notably, PSNR-avg is the average of the PSNR calculated for each of the three separate views or planes (PSNR-1, PSNR-2, and PSNR-3). This means that the PSNR is calculated independently for each view and then averaged to obtain a single value that represents the overall PSNR across all the views. PSNR-3D, in contrast, computes the PSNR for the entire 3D volumetric data as a whole. It directly compares the entire 3D real CT data to the generated 3D CT data and calculates the PSNR in one go without separating the data into individual views or planes. SSIM was also employed to evaluate the model, which quantifies the perceived visual quality of the generated synthetic CT scans. The SSIM was 0.468. The results are presented in Table 1.

In order to assess the robustness of the X2CT-GAN model under varying imaging conditions, we conducted an analysis to explore how the model’s performance is affected by deviations from the standard 90-degree orientation between the anteroposterior and lateral X-ray images. This investigation is crucial for understanding the model’s applicability in real-world clinical settings, where ideal imaging angles may not always be achievable. Table 2 presents the results of this sensitivity analysis. The evaluation metrics were compared across different angles: the standard 90 degrees, and hypothetical deviations to 85, 80, and 75 degrees. The trends observed in the table indicate a gradual decrease in model performance as the angle between the X-ray planes deviates from the orthogonal setup. This can be attributed to the reduction in the combined informative content of the X-rays as the angle diminishes. When the X-rays are orthogonal, they provide the most comprehensive and distinct information about the anatomy from two different perspectives. As the angle decreases, the overlap in visual information increases, leading to less distinct data for the model to utilize, which likely contributes to the observed decrease in performance metrics. These findings suggest that while the model demonstrates robustness to slight deviations from the 90-degree standard, its performance is significantly impacted as these deviations increase, underlining the importance of maintaining as close to an orthogonal orientation as possible for this model.

### 3.2. Qualitative Results

**Figure 4:**
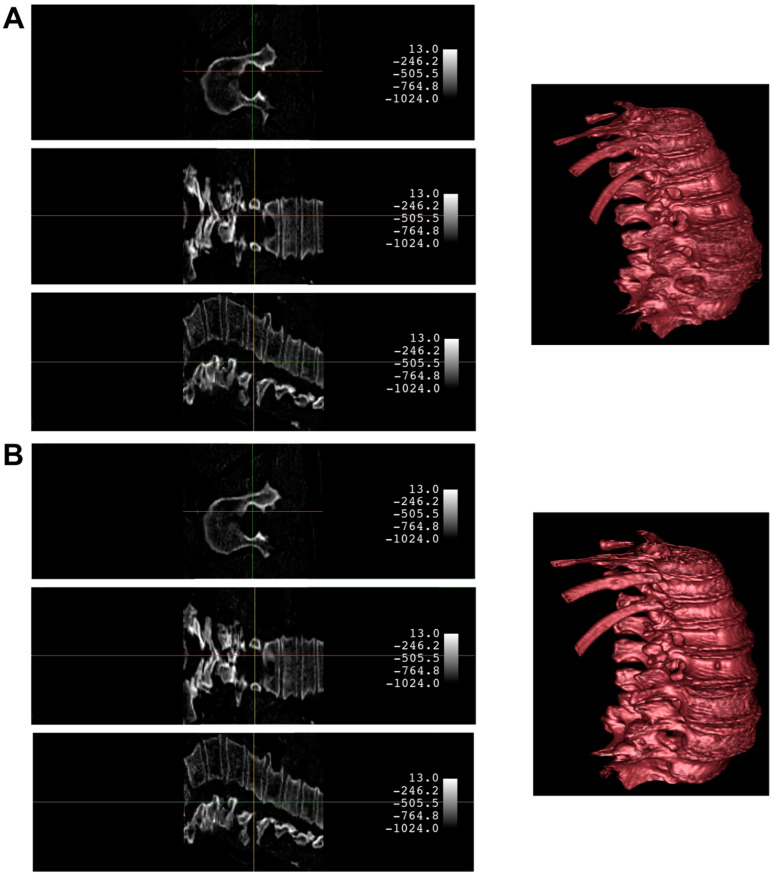
Comparison of a case with a satisfying synthetic CT quality produced by the X2CT-GAN (Panel **A**) against the ground truth CT (Panel **B**). Both panels present the 3D model and corresponding planar slices. The 3D reconstructions were generated using the CT bone preset in 3D Slicer upon importing the volumetric data. The crosshair indicates the position across different planes.

**Figure 5:**
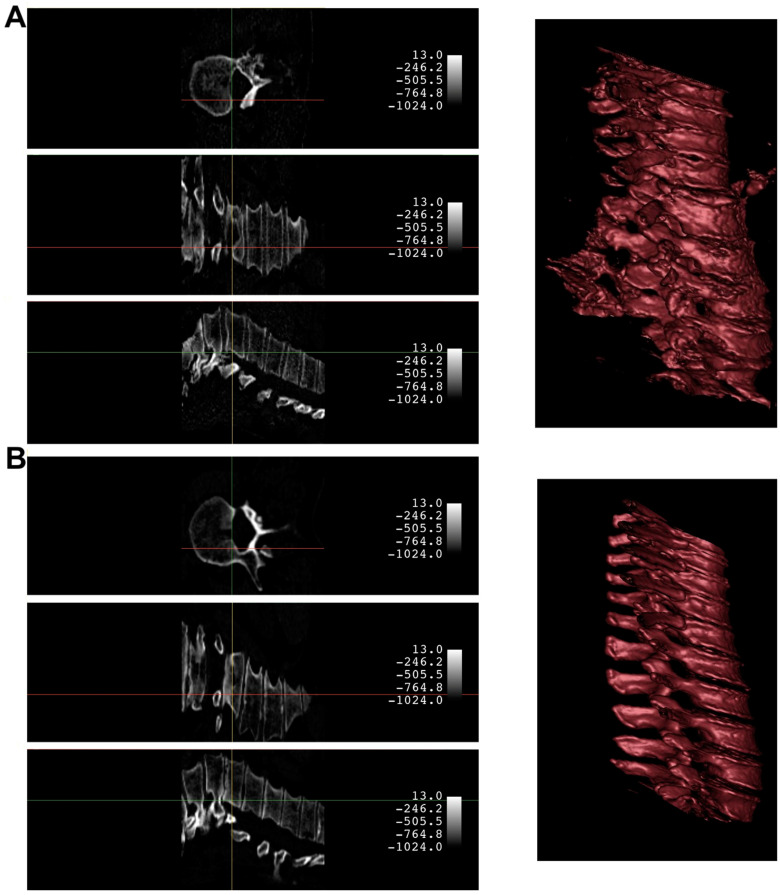
Comparison of a case with suboptimal synthetic CT quality produced by the X2CT-GAN (Panel **A**) against the ground truth CT (Panel **B**). Both panels present the 3D model and corresponding planar slices. The 3D reconstructions were generated using the CT bone preset in 3D Slicer upon importing the volumetric data. The crosshair indicates the position across different planes.

An example of the synthetic CT generated from a biplanar X-ray input is shown in Figure 4. The results were rated qualitatively by importing the real CT and synthetic CT into 3D Slicer. Although the synthetic CT provided an impressive 3D model of the spinal vertebrae based on only 2D inputs, there were structural differences in the form of the vertebrae and artificial-looking surfaces of the bone. Especially, fine structures of the bone were not appropriately reflected. Furthermore, in 14 cases, the model faced significant challenges in fully capturing the macroscopic bone structure (Figure 5). This suggests an opportunity for enhancing the model’s performance by expanding the input dataset to encompass a wider variety of input datasets commonly encountered in clinical practice.

The quantitative evaluation metrics also provide useful insights into the qualitative differences between the ground truth and synthetic CT volumes. The MAE between the synthetic and ground truth CTs was found to be 85.359, indicating that there are noticeable deviations in the intensities of the voxels. The MSE value of 31,439.027 further supports this observation, suggesting that the errors are not only localized but also significant in some areas. The PSNR values obtained for the individual channels (PSNR-1, PSNR-2, and PSNR-3) were 28.817, 28.764, and 27.599, respectively, with an average PSNR (PSNR-avg) of 28.394. These values signify that the overall contrast and dynamic range of the synthetic CT are relatively close to the ground truth. However, the lower PSNR value for the third channel indicates that some parts of the synthetic CT may have less accurate contrast and intensity representation compared to the original CT. This could be attributed to the insufficient capture of fine bone structures, leading to the artificial appearance of bone surfaces. The SSIM was 0.468. This value implies that the structural similarity between the synthetic and ground truth CTs is only moderate, reflecting the differences in the form of the vertebrae and the artificial-looking surfaces of the bone. The moderate cosine similarity value of 0.484 also suggests that the overall orientation and shape of the fine structures within the synthetic CT might not be accurately represented.

In summary, while the synthetic CT generated by the X2CT-GAN demonstrates a remarkable capability to create 3D spinal vertebrae models from synthetic 2D biplanar X-ray inputs, there are still some limitations in accurately capturing the fine bone structures and maintaining the precise morphology of the vertebrae. The artificial appearance of the bone surfaces and the differences in contrast and intensity representation in some areas indicate that further improvements to the X2CT-GAN model are necessary to achieve a more accurate and consistent performance in generating synthetic CTs from biplanar X-ray inputs.

**Figure 6:**
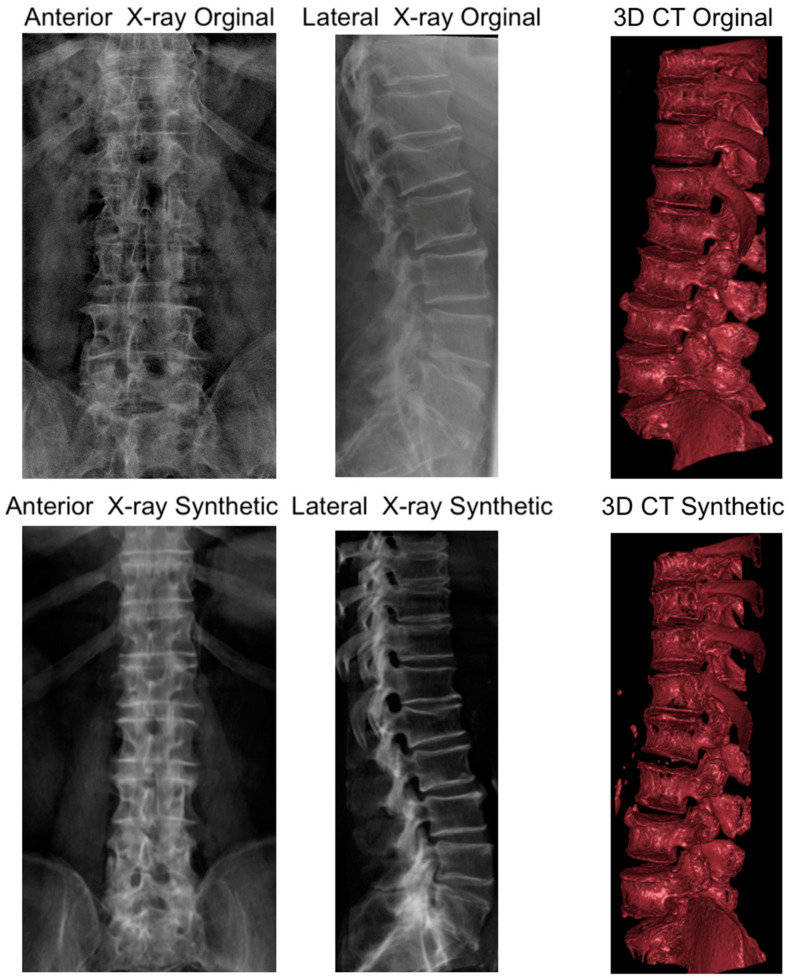
Illustration of a case with original biplanar X-ray and CT. The figure allows for a visual comparison of original and synthetic imaging data. The top row displays the original anteroposterior and lateral X-ray images alongside the original 3D CT reconstruction. The bottom row presents synthetic anteroposterior and lateral X-ray images generated from the original CT data, and a synthetic 3D CT reconstruction generated from the original biplanar X-rays. This comparison illustrates the high quality of the synthetic X-ray images relative to the originals and highlights the areas for improvement in the synthetic CT reconstruction.

The validation efforts revealed that the synthetic X-rays generated from the original CT data closely resembled the actual biplanar X-rays (Figure 6), confirming the efficacy of the preprocessing and synthetic image generation methods. However, the synthetic CT constructed from the original biplanar X-rays did not achieve the same level of quality, appearing suboptimal when compared to the original CT. This discrepancy in quality is likely attributable to the model being trained exclusively on synthetic X-rays, suggesting a need for further data augmentation with cases that have CT and original X-ray data to enhance the model’s ability to reconstruct CTs with higher fidelity.

## 4. Discussion

In this study, we applied a GAN framework to reconstruct 3D spinal vertebrae structures from synthetic biplanar X-ray images, specifically focusing on anterior and lateral views. The results demonstrated the effectiveness of the approach in reconstructing 3D spinal vertebrae structures from biplanar X-rays, although some limitations in accurately capturing the fine bone structures and maintaining the precise morphology of the vertebrae were present.

GANs, first introduced by Goodfellow et al. in 2014 [21], have revolutionized the field of deep learning by offering a novel approach to unsupervised learning. GANs consist of two neural networks, a generator, and a discriminator, which compete against each other in a game-theoretic framework. The generator learns to create realistic synthetic data samples, while the discriminator learns to distinguish between real and generated samples. Through this adversarial process, the generator progressively improves its ability to generate more convincing data [22]. This study applied the X2CT-GAN architecture as introduced by Ying et al. [11] with a novel approach of focussing on segmented regions of interest for synthetic 3D reconstruction. In comparison to previous GAN-based methods, the X2CT-GAN offers significant improvements. One key enhancement is the incorporation of a feature fusion technique that effectively combines information from multiple X-ray views, enabling a more accurate 3D reconstruction. Additionally, the architecture optimizes the generator network using a combination of MSE loss and adversarial loss, resulting in higher-quality synthetic 3D images with better structural consistency and finer anatomical details. In the medical imaging domain, GANs have shown promise in a variety of applications, including data augmentation, image synthesis, and image-to-image translation [23]. The ability of GANs to generate high-quality synthetic images has been particularly valuable in addressing challenges related to the limited availability of labeled data, data privacy concerns, and the need for multi-modal image synthesis [24]. Recent advancements in GAN architectures, such as conditional GANs [25], have further expanded the scope of applications in medical imaging. By incorporating additional information as input, conditional GANs can be trained to generate images with specific desired characteristics, making them well-suited for tasks such as 3D image reconstruction from 2D projections. Studies exploring the use of GANs for 3D image generation from chest 2D X-ray projections have demonstrated their potential in bridging the gap between conventional X-ray imaging and CT scans [11]. In this context, the application of GANs for 3D spinal vertebrae reconstruction from biplanar X-rays represents a promising direction in leveraging the power of deep learning to enhance diagnostic capabilities while reducing the cost and radiation exposure associated with traditional CT scans.

In comparison to previous work, such as the study by Humbert et al. [26], which focused on 3D reconstruction of the spine from biplanar X-rays using parametric models, our approach leverages the X2CT-GAN model for a similar purpose but with distinct methodological advancements and evaluation metrics. Humbert et al.’s study reported a mean shape accuracy of 1.3 mm and 1.0 mm for their two levels of reconstruction, respectively, which is comparable to the performance metrics observed in our study (e.g., an MAE of 0.034 per slice, and an overall PSNR of 28.394). However, our study extends the scope by utilizing a deep learning approach, which allows for more automated and potentially scalable solutions for spinal imaging. Furthermore, while Humbert et al.’s method required manual adjustments for higher accuracy, our X2CT-GAN model automates the generation of 3D spinal reconstructions, potentially reducing the time and expertise required for analysis. This contrast highlights the evolution of 3D spinal imaging techniques, moving from semi-automated parametric models to fully automated deep learning systems. Nevertheless, the slightly higher MAE and lower PSNR in our study compared to the high accuracy of Humbert et al.’s second level of reconstruction (mean shape accuracy of 1.0 mm) suggest that while our model offers advantages in terms of automation and efficiency, there is still room for improvement in achieving the utmost precision, especially in capturing fine bone structures and maintaining accurate morphology. Our study’s use of a GAN-based method for 3D reconstruction of spinal structures from bi-planar X-rays offers insights that complement and contrast with similar recent works, such as the study by Yang et al. Yang et al. focused on improving the structural accuracy of their reconstructions, as evidenced by their use of metrics such as the Dice similarity coefficient and the structural similarity index. They reported high values in these metrics, indicating a strong structural resemblance to the ground truth. This contrasts with our study, which prioritized intensity accuracy and noise performance, reflected in our selection of metrics such as MAE, MSE, and PSNR. While both studies highlight the potential of GAN-based methods in medical imaging, they each offer a unique perspective on model performance. Our approach, by focusing on metrics such as MAE and PSNR, emphasizes the fidelity of voxel intensities and noise characteristics, which are crucial in certain clinical scenarios where precise intensity values are key to diagnosis and treatment planning. The divergence in the methodological and evaluative choices underlines the multifaceted nature of 3D reconstruction research. It demonstrates that various aspects of model performance are prioritized differently depending on the specific clinical or research question being addressed. This variation in focus is not just a reflection of the diverse capabilities of GAN-based approaches, but also an indication of the broad range of clinical needs and research objectives within medical imaging.

Recent advancements in the field, as exemplified by studies such as the “BX2S-Net” [27] and PerX2CT’ [28], provide further context for our work’s positioning within the evolving landscape of 3D medical imaging. Notably, our study was completed before the introduction of these developments, limiting the possibility of sophisticated comparisons. The “BX2S-Net” framework, with its dimensionally consistent architecture and feature-guided decoding, and “PerX2CT”, with its focus on perspective projection and computational efficiency, have built upon and extended the principles we explored. Our work primarily focused on intensity accuracy and noise performance, providing a critical stepping stone for future studies. The different emphases of BX2S-Net on edge region quality and multi-view fusion, and PerX2CT on computational speed, highlight the continuous innovation in this field. These recent studies underline the dynamic nature of 3D reconstruction technology and emphasize the importance of evolving model accuracy alongside computational efficiency. X2CT-GAN, in establishing the potential and laying the groundwork for GAN-based spinal reconstruction from 2D X-rays, might been instrumental in guiding future comparisons and innovations. In conclusion, our study not only showcased the capabilities of GAN-based methods in medical imaging but also set the stage for the rapid advancements that will follow. This ongoing evolution in 3D reconstruction technologies both validates and extends the value of our work. As the field continues to progress, our study serves as a significant reference point, highlighting foundational concepts that might inform and inspire new directions in research and development in this field.

Notably, the objective of this work is not to supplant CT scans with biplanar X-rays. Although our proposed method can reconstruct the overall structure of spinal vertebrae, finer anatomical details may still exhibit some artifacts, as seen in our results. Nevertheless, the developed approach has the potential to find specialized applications in clinical practice, especially when trained with larger datasets and more advanced techniques such as cross-modality transfer learning. For instance, the approach could be employed to measure the dimensions of spinal vertebrae, assess vertebral alignment, or detect anatomical abnormalities in the reconstructed 3D volume on a macroscopical scale. Moreover, the method may be utilized for treatment planning in radiation therapy, preoperative planning, and intra-operative guidance during minimally invasive spinal procedures. Further, this technique can be used for educational purposes for students and residents when only biplanar X-rays of patients are available. As a valuable addition to low-cost X-ray machines, this technique can provide healthcare professionals with an artificial CT-like 3D volume of spinal vertebrae, offering clinical insights with reduced cost and radiation exposure.

An important aspect of future work revolves around the model’s training with real patient data, specifically incorporating both CT images and corresponding biplanar X-rays. The current limitations observed in accurately capturing fine bone structures and maintaining precise morphology could be significantly mitigated by training the GAN model on a more diverse dataset that includes actual patient images. Such a dataset would ideally encompass a wide array of patient X-rays taken under various conditions, reflecting the real-world variability in angles and orientations. Additionally, our study’s reliance on the COLONOG subset of the CTSpine1K dataset, primarily representing non-pathological spinal states, highlights the necessity for a more inclusive dataset in future research. The inclusion of cases with various spinal pathologies would be invaluable. This expansion would not only allow for a more comprehensive assessment across different spinal conditions but also enhance the model’s diagnostic applicability in detecting and analyzing spinal anomalies or pathologies. While the current dataset provided a solid foundation for initial model training and validation, the integration of a broader spectrum of spinal health states—ranging from normal to various pathological conditions—is crucial for the model’s evolution and relevance in clinical diagnostics. This development would be a substantial step forward in the application of deep learning techniques in medical imaging, particularly in environments with limited access to advanced imaging technologies. The robustness and accuracy of the model, when trained on such diverse and clinically representative data, would significantly elevate its potential in practical medical applications.

Additionally, we need to address the resolution of the output images produced by our model. The chosen resolution of 128 × 128 × 128 voxels with an output voxel size of 1 mm × 1 mm × 1 mm was selected to strike a balance between image detail and computational demands. This resolution, while on the coarser end, remains within the clinical range and is particularly suitable for macroscopic analyses where a higher level of detail may not substantively change clinical outcomes. However, we acknowledge that certain clinical applications may benefit from higher-resolution imaging. To approximate the standard clinical resolution of approximately 0.625 mm, a significant increase in computational resources would be required [29,30]. Doubling the resolution in each dimension to achieve this finer scale would result in an eightfold increase in the total number of voxels, necessitating advanced computational hardware with increased memory capacity and processing power. Such an upgrade would allow for processing larger volumes of data and maintaining efficient reconstruction times, yet it must be justified against the clinical value gained versus the additional computational expense incurred. As we look to the future, the evolution of hardware capabilities and the optimization of deep learning algorithms are likely to provide opportunities for achieving higher resolutions. The burgeoning field of cloud computing and specialized AI processing units also presents a promising avenue for decentralizing and thus democratizing access to the computational power necessary for high-resolution 3D reconstruction.

## 5. Conclusions

In summary, while the synthetic CT generated by the X2CT-GAN demonstrates a remarkable capability to create 3D spinal vertebrae models from synthetic 2D biplanar X-ray inputs for use in many areas, there are still some limitations in accurately capturing the fine bone structures and maintaining the precise morphology of the vertebrae. The artificial appearance of the bone surfaces and the differences in contrast and intensity representation in some areas indicate that further improvements to the model are necessary in order to achieve a more accurate and consistent performance in generating synthetic CTs from biplanar X-ray inputs. Future work could focus on refining the model architecture, applying cross-modality learning, and utilizing novel loss functions to enhance the overall quality of the synthetic CT volumes.

## Figures and Tables

**Table 1:** Evaluation metrics (validation dataset; n = 88) for the proposed X2CT-GAN approach to generate synthetic 3D models of the spinal vertebrae from biplanar X-ray inputs.

Metrics	Value
MAE0	0.0342
MSE0	0.005
MAE	85.359
MSE	31,439.027
Cosine Similarity	0.4840
PSNR-3D	27.432
PSNR-1	28.812
PSNR-2	28.764
PSNR-3	27.599
PSNR-avg	28.394
SSIM	0.468

**Table 2:** Evaluation metrics for the proposed X2CT-GAN approach to generating synthetic 3D models of the spinal vertebrae from biplanar X-ray inputs, showing the impact of varying the angle between anteroposterior and lateral X-rays. The table compares the model’s performance on the validation dataset (n = 88) at standard 90-degree orientation with hypothetical performance at 85, 80, and 75 degrees, illustrating the sensitivity of the model to deviations from orthogonal biplanar imaging.

Metrics	90 Degree	85 Degree	80 Degree	75 Degree
MAE0	0.0342	0.0353	0.0376	0.041
MSE0	0.005	0.005	0.006	0.008
MAE	85.359	86.508	88.177	90.284
MSE	31,439.027	32,155.013	32,311.141	33,409.422
Cosine Similarity	0.4840	0.475	0.465	0.450
PSNR-3D	27.432	27.294	26.924	26.559
PSNR-1	28.812	28.749	28.614	28.484
PSNR-2	28.764	28.652	28.554	28.356
PSNR-3	27.599	27.424	27.268	27.023
PSNR-avg	28.394	28.251	28.194	27.996
SSIM	0.468	0.461	0.453	0.446

## Data Availability

Publicly available datasets were analyzed in this study. This data can be found here: https://github.com/Freiburg-AI-Research (accessed on: 15 August 2023).
